# Properties and nitrate leaching mitigation effect of thermally treated biomass-a case study of tropical and subtropical islands

**DOI:** 10.1038/s41598-026-41496-1

**Published:** 2026-03-03

**Authors:** Kosuke Hamada, Satoshi Nakamura, Takahiro Yoshida

**Affiliations:** 1 Tropical Agriculture Research Front, International Research Center for Agricultural Sciences, Okinawa, Japan; 2 Crop, Livestock and Environmental Division, International Research Center for Agricultural Sciences, Tsukuba, Japan; 3 Forest Research and Management Organization, Tsukuba, Japan

**Keywords:** Nitrate leaching, Nitrate adsorption, Torrefied biomass, Biochar, Acidic soil, Ecology, Ecology, Environmental sciences

## Abstract

This study assessed the properties of thermally treated biomass including torrefied biomass and biochar and their effect on nitrate leaching mitigation. The raw biomass materials were derived from herbaceous sugarcane residue (bagasse) and woody Alexandrian laurel, which are common feedstocks in subtropical and tropical islands. The biomass materials were treated at various temperatures ranging from 220 to 800 °C. Nitrate adsorption capacity and mitigation effect on nitrate leaching when it was applied in acidic soil were evaluated. We observed typical changes in the yield and functional groups of the thermally treated biomass with increasing treatment temperature. Nitrate adsorption tended to occur with 200–300 °C materials at a rate of approximately 3.0–7.0%. Nitrate leaching decreased only with the 800 °C material, with a mitigation rate of 30%. The experiments and multiple regression analysis revealed that the low pH and hydrophilicity of materials < 300 °C positively affected nitrate adsorption, whereas the high surface area of the 800 °C material significantly reduced nitrate leaching. Further investigation of the long-term effects of thermally treated biomass application at different treatment temperatures is necessary to reduce the nitrate leaching on subtropical and tropical islands.

**Supplementary Information:**

The online version contains supplementary material available at 10.1038/s41598-026-41496-1.

## Introduction

Since the Haber–Bosch process was developed, the amount of chemical fertilizer production along with food production has increased, thereby supporting the world population^1,2^. However, the excessive use of chemical fertilizers leads to reduced efficiency in nitrogen use^3^, causing environmental problems such as water and air pollution along with water eutrophication^4^. The anthropogenic production of reactive nitrogen has increased almost five-fold compared with that in 1960^5^, and the use of reactive nitrogen is expected to increase^6^, indicating that reactive nitrogen-related problems will be severe.

Nitrate leaching is a serious problem in subtropical and tropical islands^7,8^. The soil is generally weathered, acidic, and has low fertility^9–11^, requiring a high amount of chemical fertilizer for crop production. The improvement of soil fertility is generally difficult because organic matter quickly decomposes under high humidity and temperature conditions^12^. This accelerates nitrate leaching, thereby degrading a unique ecosystem^13,14^, making the islands famous for tourism. In short, leaching harms the environment and economies of the islands. Moreover, poor resource recycling presents a problem for these islands. For instance, the manure use rate on a subtropical island in Japan is < 15%^15^, and sugarcane residue (bagasse) is left over. These organic resources should be applied to agricultural fields to reduce over-dependence on chemical fertilizer. Therefore, an initiative involving the change of resources to useful materials is required, and a thermally treated material is a promising initiative.

Biochar and torrefied biomass are made from biomass by heat treatment above or below 300 °C, respectively, under limited oxidant concentrations. Biochar, produced by the carbonization of biomass, contributes to carbon sequestration through its application to agricultural fields. Moreover, its porous structure entraps nitrate^16^, thereby reducing nitrate leaching^17–20^. However, the mass yield of biochar is very low at approximately 25% compared to dry feedstock^21,22^. Torrefied biomass has been used in the energy sector^23,24^. The biomass is considered more cost-effective and environmentally friendly than biochar owing to its relatively low energy requirement^25^. Recently, the material has garnered increasing attention for agricultural use. For example, the application of torrefied biomass to agricultural fields can positively alter soil physicochemical properties, crop growth, and GHG emissions^26^. Moreover, it retains organic matter that decomposes gradually^27^, thus possibly improving soil fertility in subtropical and tropical islands. As written above, numerous aspects of the application effect of torrefied biomass have been evaluated; however, very few studies focus on nitrate leaching and adsorption.

The properties of thermally treated materials differ depending on feedstock. Sugarcane residue is common in subtropical and tropical islands. Several studies have evaluated the effect of sugarcane residue biochar on nitrogen load^28,29^. However, studies that have used the residue are limited. Moreover, a relatively wide treatment temperatures should be considered to identify optimal materials for nitrogen load mitigation. This study was conducted by Cheng et al.^30^, who applied 300, 500, and 800 °C wood materials to subtropical forest soil. However, a more detailed evaluation is lacking, particularly in the lower temperature range (< 300 °C) where material properties change drastically. This lack of information hinders the expansion of the application of thermally treated biomass in subtropical and tropical islands.

With the ultimate goal of reducing the nitrogen load in tropical and subtropical islands through the application of thermally treated biomass, we evaluated changes in its properties and nitrate adsorption capacity with treatment temperature (220–800 °C). The effect on reduction in nitrate leaching was also evaluated by mixing with acidic soil, which is abundant on these islands. We conducted the study in a subtropical island in Japan and used herbaceous and woody biomass, which are common in the island.

## Methods

### Preparation of materials

The study was conducted in Japan International Research Center for Agricultural Sciences (JIRCAS) in Ishigaki Island, Japan (24°22ʹ43ʺ N; 124°11ʹ43ʺ E). The island has huge sugarcane (*Saccharum officinarum*) plantations and various local trees, such as Alexandrian laurel (*Calophyllum inophyllum*, locally called as “*Terihaboku*” or “*Yarabu*”), as shelterbelt. Sugarcane residue (bagasse) and Alexandrian laurel wood branches, which are produced through tree maintenance, are abundant and not fully reused inside the island. There is urgent need to turn them into valuable products or apply them to agricultural lands. For this reason, we used bagasse and Alexandrian laurel as feedstock.

First, both feedstocks were coarsely crushed and dried. The moisture contents were approximately 10% and 24% for bagasse and Alexandrian laurel, respectively. The bagasse used for the 800 °C treatment was further milled, then compacted into pellets, 6 mm in diameter. Notably, because Fourier Transform Infrared Spectroscopy (FTIR; IRSpirit; Shimadzu Corporation, Kyoto, Japan) results of the pelletized and unpelletized 800 °C biochar were identical, we considered the effect of pelletization on biochar quality to be slight.

Heat treatments were performed within the range of 220–800 °C using electric furnaces in the laboratory scale. Treatments were performed at temperatures of 220, 240, 270, 300, 400, and 800 °C for bagasse and 245, 295, 345, 500, and 800 °C for Alexandrian laurel. An inert gas oven (DRJ463WA, Advantec toyo kaisha, Ltd., Tokyo, Japan) was employed at temperatures below 350 °C, and a muffle furnace (FUW232PA, Advantec toyo kaisha, Ltd.) was employed for treatments exceeding 400 °C. In the procedure with the inert gas oven, the samples were placed on stainless plates, then the oven was heated under the average heating rate of 8 °C min^− 1^ with a nitrogen (99.995%) flow of 30 L min^− 1^.After holding the torrefaction temperature for approximately 5 min, the oven was slowly cooled under a nitrogen atmosphere at an average rate of 1 °C min^− 1^ to room temperature^31^. The procedure using the muffle furnace was as follows. First, the samples were placed in aluminum containers (at 400 and 500 °C) or in alumina containers (at 800 °C), and sealed with a lid to prevent spontaneous combustion. These were then placed in the oven, and the oven was heated under 10 L min^− 1^ of nitrogen flow atmosphere. The oven was heated at a rate of approximately 5 °C min^− 1^ and maintained around 100 and 300 °C for approximately 1 h to remove volatiles before reaching the treatment temperature. The treatment temperature was maintained for 90–120 min. Then the oven was gradually cooled to room temperature at an average rate of 1 °C min^− 1^ under a nitrogen atmosphere. before the treated sample was removed.

All materials were finely ground (< 1 mm) for further experiments. Samples hereafter refer to ground materials. To evaluate the mass loss profile on heating, measurements were performed using a thermobalance (TG-DTA8122; Rigaku Holdings Corporation, Tokyo, Japan) at a rate of 10 °C min^− 1^ under a nitrogen atmosphere. Approximately 10 mg of the sample was employed for the thermobalance measurements. The surface functional groups were evaluated using FTIR. The results were described in the Kubelka–Munk index.

### Properties of the materials

Fixed carbon content was determined using the following Eq. 

1 $$\:FC=100-\left(VM+Ash\right)$$

where *FC* is fixed carbon (wt% on dry basis), *VM* is volatile matter (wt% on dry basis), and *Ash*: Ash content (wt% on dry basis). Ash and VM were measured according to ISO 18,122 and ISO 18,123 standards^32,33^, respectively. VM was determined from the mass loss of a 1 g sample placed in a quartz container with a lid, placed in a muffle furnace, and pyrolyzed at 900 °C for 7 min. The ash content was determined by weighing the samples after 2 h of ignition at 815 °C using a muffle furnace.

The contents of carbon, hydrogen, and nitrogen in each sample were measured using the CHN coder MT-6 (Yanaco Technical Science, Co., Ltd., Tokyo, Japan). The pH and electric conductivity (EC) of material were measured using the glass electrode method and a LAQUA F-72 (HORIBA Advanced Techno, Co., Ltd., Kyoto, Japan) and EC meter ES-51 (HORIBA Advanced Techno, Co., Ltd.) after extraction in a 1:5 sample–water ratio with 2 h equilibration time. The specific surface area and pore distribution of each sample were measured using the BET method using the same samples (NOVA touch 4Lx; Anton Paar GmbH, Graz, Austria). Prior to the measurement in the BET method, degassing was conducted for 12 h at 70 °C for the raw materials and 6 h at 110 °C for the others, respectively. The pH and EC measurement was conducted with 2 replicates and the other evaluations were performed without replicates.

### Nitrate adsorption test

The test design was based on the design outlined by Kameyama et al.^34^. In a syringe, each material (0.5 g) and KNO_3_ solution (40 mL, 100 mg N L^− 1^, pH = 9.0) were added, and the mixture was shaken for 24 h in triplicate. The shaking speed ranged from 120 to 130 rpm. The solution was then filtered (ADVANTEC Filter paper 6; Toyo Roshi Kaisha, Ltd.) and nitrate concentration and pH of the solution were measured using an Auto Analyzer III (BL TEC K. K., Tokyo, Japan) and LAQUAF-72, respectively. The nitrate adsorption rate was calculated as follows.

2 $$\:Rate=\:\left(1-{N}_{end}/{N}_{ini}\right)\times\:100$$

where *Rate* is the nitrate adsorption rate (%), *N*_*end*_ is the nitrate concentration in the solution after shaking (mg L^− 1^), and *N*_*ini*_ *is* the initial nitrate concentration in the solution (mg L^− 1^). To consider nitrate desorption, the same protocol was followed using deionized water. The desorption was then added to the initial concentration.

### Nitrate leaching test

We conducted a test based on the study by Kameyama et al.^34^. Each material was mixed in air-dried soil at a ratio of 2% by dry weight. Three replicates were used for each experiment. The saturated hydraulic conductivity of the soil was determined by filling 250 mL cylinders with soil under the same conditions as the pipe-filled soil using KSAT (METER Group, Pullman, WA, USA) (three replicates for each condition). The applied soil was obtained from depths ranging from 50 to 100 cm of a field of JIRCAS, and is classified as Dystric Cambisol according to the World Reference Base for Soil Resources 2014^35^. Its properties are listed in Table 1. The pH of mixed soil was measured using the same procedure as the pH measurement of materials. Vinyl chloride pipes with a diameter of 7 cm and height of 20 cm were uniformly filled with the mixed soil. The soil dry density was 1.25 g cm^− 3^ and the height of the soil column was 15 cm. The pipe had a fully-opened valve at the bottom for drainage sampling. First, the column was capillary-saturated with deionized water for 24 h. Then, 700 mL (two pore volumes) of deionized water was added to the soil surface to replace pore water in the sample.

**Table 1:** Chemical and physical properties of the soils.

pH	EC	Bray1–*P*	Bray2–*P*	NO_3_–*N*	NH_4_–*N*	T–*N*	T–C		
(H_2_O)	mS m^− 1^	mg kg^− 1^				g kg^− 1^			
5.25	4.28	4.2	25.81	1.29	5.81	0.65	3.59		
Exchangeable cations									
Ca	k	Mg	Na	CEC	Sand	Silt	Clay	Texture	K_sat_
cmolc kg^− 1^				cmolc kg^− 1^	%				cm s^− 1^
2.05	0.13	0.83	0.13	8.52	41	10.3	41	Heavy clay	5.6 × 10^− 3^

K_sat_ is saturated hydraulic conductivity.

Using a peristaltic pump, 100 mL of KNO_3_ solution (100 mg N L^− 1^) was given six times, followed by six times with 100 mL of deionized water application. The application time was approximately 20 min. We collected the drainage from the bottom after every application and measured the amount. We then measured nitrate concentration in each drainage using an Auto Analyzer III.

The test duration of adsorption and leaching tests was shorter than two days, including pre-treatment. We, therefore, disregarded the mineralization of organic matter in the materials. Dunnett’s test was conducted in the adsorption and leaching tests to evaluate the significance of differences from the control with a significance threshold of *p* < 0.05 and *p* < 0.01.

### Multiple regression analysis

Multiple regression analysis was applied to determine the parameters that influenced the results of nitrate adsorption and leaching tests. The adsorption rate was set as the objective parameter for the adsorption test. For the leaching test, we set the time required to reach the maximum leaching rate as the objective parameter. To obtain time, we applied the Gompertz model to the observed temporal changes in cumulative leaching. The Gompertz model is a sigmoidal curve, similar to that of the logistic model. The Gompertz model depicts an asymmetrical curve, whereas the logistic model depicts a symmetrical curve^36^. The change in total cumulative leaching in this study was an asymmetrical curve; therefore, we deemed the Gompertz model suitable. The Gompertz model is expressed as follows.

3 $$\:Y=a\times\:{e}^{-{e}^{\left(b-cx\right)}}$$

where *Y* is the cumulative leaching (mg), *x* is the time (min), a is the maximum potential leaching rate (mg min^− 1^), *b* is the intercept, and *c* is the leaching rate. The maximum leaching rate during the experiment was determined by *ac*/exp and the time (min) at this point was determined by *b/c*.

## Results and discussion

### FTIR results

**Fig. 1:**
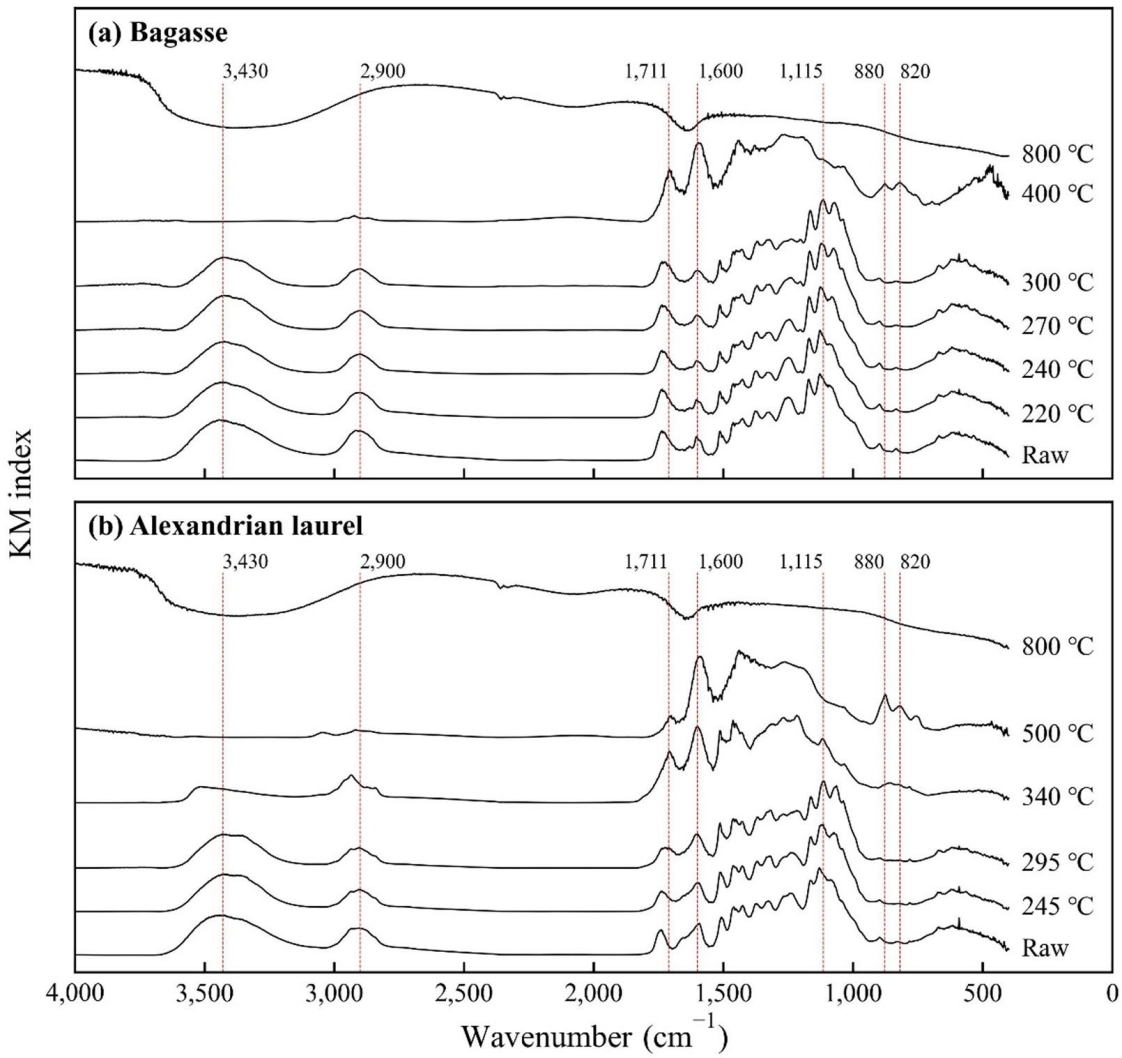
FTIR results of bagasse and Alexandrian laurel materials. FTIR and KM mean Fourier Transform Infrared Spectroscopy and Kubelka–Munk, respectively.

For both materials, the peaks observed in the raw material decreased with increasing temperature to 300 °C (e.g., 1,115, 2,930, and 3,470 cm^− 1^; Fig. 1). These peaks indicate the C–O–C and C–H bonds of the alkyl groups and –OHs, respectively. The decrease in the intensity of the peaks was particularly large for the C–H and –OH groups and was more gradual in Alexandrian laurel than that in bagasse. According to Matsui et al.^37^, the main components of gas emissions up to 400 °C during heat treatment are primarily carbon and oxygen. The decrease in the –OH group in this study may have been induced by the same reason. The decrease in the –OH groups also increased hydrophobicity^38^. However, the result indicated that only the functional groups decreased and wavelengths were similar to those of the raw materials, indicating that organic matter was retained (Fig. 1). At 340–500 °C, the trend of the peaks changed significantly compared to that of the raw material; the 2,930 and 3,470 cm^− 1^ peaks, corresponding to the alkyl and hydroxy groups, decreased. Conversely, the 1,600 and 1,711 cm^− 1^ peaks, which indicate C = C and C = O stretching vibrations, increased respectively. At 400–500 °C, the peaks at 820 and 880 cm^− 1^ (both aromatic groups) increased. Therefore, in this study, the hydroxy and alkyl groups decreased, whereas the aromatic and carboxyl groups increased, indicating transition to a more stable structure. At 800 °C, no obvious peaks were observed. These changes were similar to the FTIR results of graphite from previous studies^39,40^; we considered these materials to have changed to a graphite structure.

### Changes in the properties of thermally treated biomass 

**Fig. 2:**
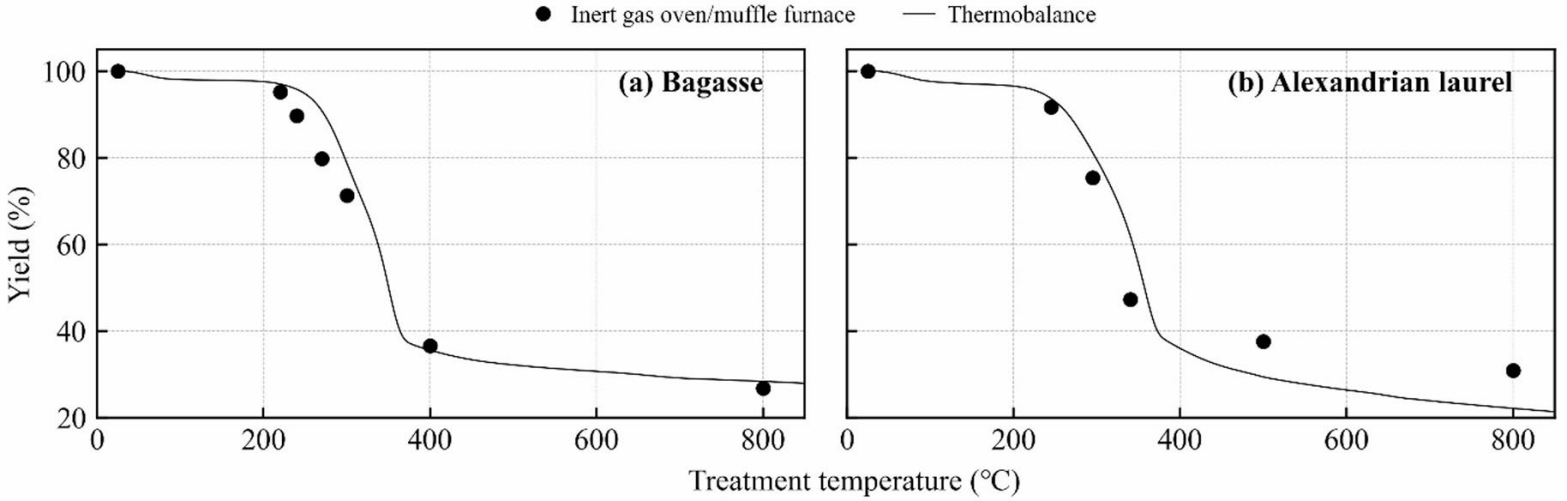
Changes in the yield of thermally treated biomass under different treatment temperatures. An inert gas oven was employed at temperatures below 350 °C, and a muffle furnace was used for treatments above 400 °C.

Yield changes in the actual sample in the inert gas oven/muffle furnace and in the thermobalance were almost identical after treatment (Fig. 2), indicating the reliability of the yield results. The yield changed drastically between 200 and 400 °C and finally gave approximately 25% at 800 °C. The FC and ash increased with increasing treatment temperature, whereas the VM content decreased (Table 2). The change was drastic above 300 °C for bagasse and 340 °C for Alexandrian laurel. The pH of bagasse material decreased to 240 °C then increased to 800 °C. For Alexandrian laurel, the pH increased as the treatment temperature increased. Below 300 °C, the pH for both materials was below 7; the materials for Alexandrian laurel had a lower pH than that of bagasse material within this temperature range. According to Matsui et al.^37^, torrefied biomass and low-temperature biochar exhibit acidity owing to the presence of numerous carboxyl and phenolic hydroxy groups, whereas high-temperature biochar shows alkalinity owing to the decrease in these functional groups and presence of ash, which contains metal oxides. The carbon content increased as the treatment temperature increased, whereas hydrogen and nitrogen contents decreased. The changes in the carbon and nitrogen contents were moderate up to 300 °C. At low temperatures, tar accumulates on the material surface and volatilizes with an increase in treatment temperature^41^. In these processes, the carbon and nitrogen contents appeared stable. After tar volatilization, the carbon content increases, whereas the nitrogen content decreases. The same phenomenon may have occurred in this study (Table 2). The H/C ration decreased as the treatment temperature increased, indicating a decrease in the –OH group, that is, increased hydrophobicity. In the bagasse material, a large decrease was observed at above 300 °C, in Alexandrian laurel, it was observed at > 340 °C (Table 2). We considered the materials below these temperatures to be rather hydrophilic. All the bagasse materials at < 300 °C had higher H/C and O/C ratios than that of Alexandrian laurel at 245 °C. This indicated that bagasse materials were more hydrophilic than Alexandrian laurel material at these temperatures. The EC initially increased upon thermal treatment, then gradually decreased from 200 to 500 °C, after which it increased again; the highest EC was observed at 800 °C. At 800 °C, a similar trend was observed in previous studies on high-temperature biochar (e.g., Tu et al.^42^ owing to its high ash content and graphitization^37,43^. No previous studies have summarized the EC within the temperature range of 200–500 °C (particularly in the 200–300 °C range), and according to the FTIR results, there was a decreasing trend in the hydroxyl and carboxyl groups as the treatment temperature increased from 200 to 500 °C (Fig. 1). At temperatures > 400 °C, the decrease in these functional groups and formation of aromatic and carboxyl groups occurred simultaneously; however, these changes were only partially observed. This decrease in functional groups and the low ash content may induce a decrease in EC in the 200–500 °C range.

**Table 2:** Characteristics of each material.

	Ash	VM	FC	pH	EC	C	H	*N*	H/C	O^*^/C	Average pore diameter
	%	%	%	(H_2_O)	mS m^− 1^	%	%	%			nm
Bagasse	2.3	83.9	13.8	6.95	40.5	44.0	6.31	0.37	1.72	0.84	22.1
B220	2.9	82.1	15.0	6.64	58.2	45.1	6.16	0.36	1.64	0.80	21.1
B240	2.8	80.5	16.7	6.44	55.1	46.4	6.18	0.30	1.60	0.76	18.1
B270	2.8	76.7	20.5	6.41	44.3	48.4	5.91	0.31	1.46	0.70	17.8
B300	3.4	72.3	24.4	6.75	32.8	50.6	5.91	0.31	1.40	0.64	17.7
B400	6.1	24.0	69.9	7.28	28.0	68.2	3.56	0.43	0.63	0.31	10.1
B800	14.2	4.5	81.3	9.79	66.3	79.0	0.84	0.16	0.13	0.19	9.2
Alexandrian laurel	2.1	78.6	19.3	5.50	30.8	47.3	6.13	0.18	1.55	0.74	15.4
AL245	2.1	76.3	21.6	5.97	63.4	51.7	5.98	0.21	1.39	0.61	15.0
AL295	2.5	67.5	30.0	6.85	41.3	54.7	5.47	0.18	1.20	0.54	12.2
AL340	3.8	43.0	53.2	7.34	49.6	66.3	4.53	0.23	0.82	0.33	9.6
AL500	5.6	20.3	74.2	8.53	33.9	75.3	2.94	0.20	0.47	0.21	14.6
AL800	6.3	6.4	87.3	9.97	74.4	75.6	1.22	0.15	0.19	0.23	6.7

* Oxygen content was calculated by 100 – (C + H + N).

B and AL indicate materials made from bagasse and Alexandrian laurel, respectively. The numbers after B or AL indicate the treatment temperature. VM and FC indicate volatile matter and fixed carbon, respectively.

**Fig. 3:**
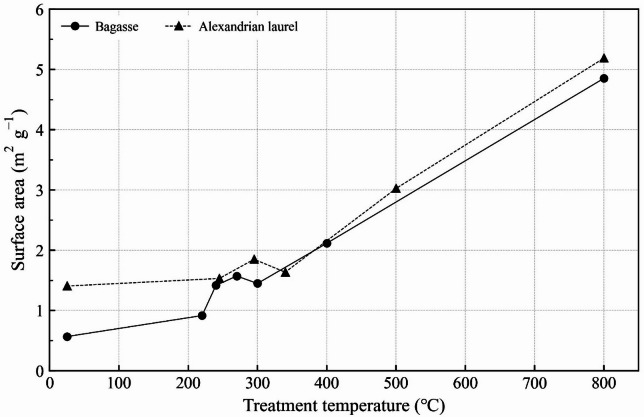
Surface area of each material.

The average pore diameter gradually decreased at temperatures below 300 °C, and above this temperature, the decrease tended to be drastic, except for the 500 °C Alexandrian laurel, which exhibited an abrupt increase. The surface area increased with the increase in treatment temperature (Fig. 3). In the raw material case, the area of Alexandrian laurel was larger than that of bagasse. However, the difference disappeared following the higher temperature thermal treatment. Through thermal treatment, the materials obtained a porous structure.

### Nitrate adsorption and leaching

**Fig. 4:**
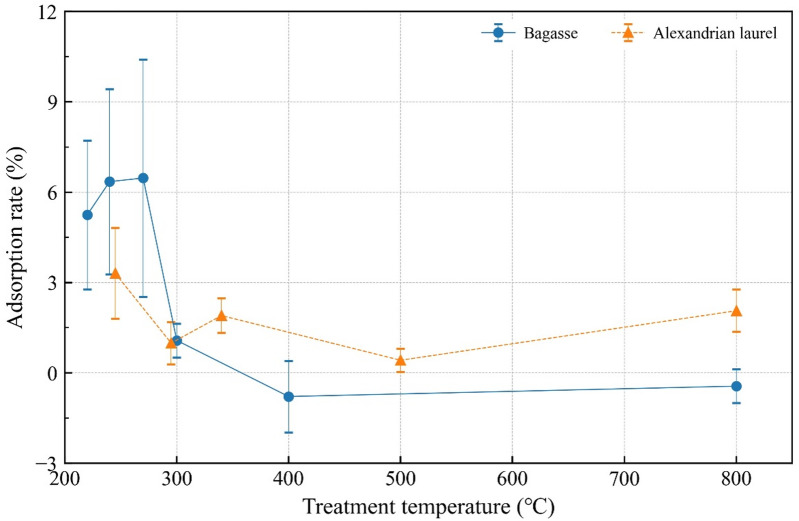
Adsorption rate of nitrate of each material. Error bars indicate standard error.

No significant difference was observed; however, both bagasse-derived and Alexandrian-laurel-derived materials showed higher adsorption rates within the torrefaction range of 200–270 °C with a rate of approximately 3.0–7.0%. (Fig. 4). The adsorption rates of bagasse were high at 220–270 °C and decreased sharply at 300 °C. The adsorption rate became negative at 400 and 800 °C, suggesting that nitrate was released from these materials. The adsorption rate of Alexandrian laurel was a maximum at 245 °C. Above this temperature, the rates decreased; they all ranged from 1 to 2%. Overall, the rates of bagasse materials exceeded those of Alexandrian laurel under 200–270 °C; however, above this temperature, the opposite trend was observed. The pH of the bagasse material dropped toward 270 °C, then rose as the treatment temperature increased (pH = 5.95–10.38; Table 3). Regarding Alexandrian laurel, the pH of 270 °C material was the lowest and it rose as the treatment temperature increased (pH = 5.13–10.00). Only 800 °C material from both materials showed higher pH than the original KNO_3_ solution. The pH was lower in Alexandrian laurel than that in bagasse at < 300 °C. Fidel et al.^44^ indicated that nitrate adsorption to biochar is pH-dependent and higher in the low pH area. In this study, the adsorption rate was higher in the low temperature area, that is, the lower pH area. The higher rate was induced by the low pH. However, the adsorption rate in Alexandrian laurel was lower, although it had a lower pH in the low temperature range (Fig. 4). Bagasse exhibited higher H/C and O/C ratios than Alexandrian laurel under low temperature treatment (< 300 °C), as shown in Table 2 and retained a stronger absorbance around 3,430 cm^− 1^ in the FTIR spectra as shown in Fig. 1. These observations indicate that bagasse contains more OH functional groups than Alexandrian laurel, which therefore holds higher hydrophilicity, and consequently contributes to the higher nitrate adsorption rate. This possibly contributed to the higher nitrate adsorption rate; however, the pH in the bagasse case was higher than that in the Alexandrian laurel case. This idea was supported by multiple regression. In the analysis, the pH of the solution and H/C ratio were selected with an adjusted R^2^ of 0.60. The p-value was < 0.05 and < 0.01 for the pH of the solution and H/C ratio, respectively. Our results indicated that the nitrate adsorption ability was stronger in materials treated at < 300 °C owing to lower pH and hydrophilicity.

**Table 3:** pH of solution in adsorption test.

B220	B240	B270	B300	B400	B800	AL245	AL295	AL340	AL500	AL800
6.63	6.17	5.95	6.28	7.06	10.38	5.13	5.41	5.64	8.68	10.00

B and AL indicate materials made from bagasse and Alexandrian laurel, respectively. The numbers after B or AL indicate the treatment temperature. Each value indicates the pH of KNO_3_ solution after 24 h-shaking.

**Fig. 5:**
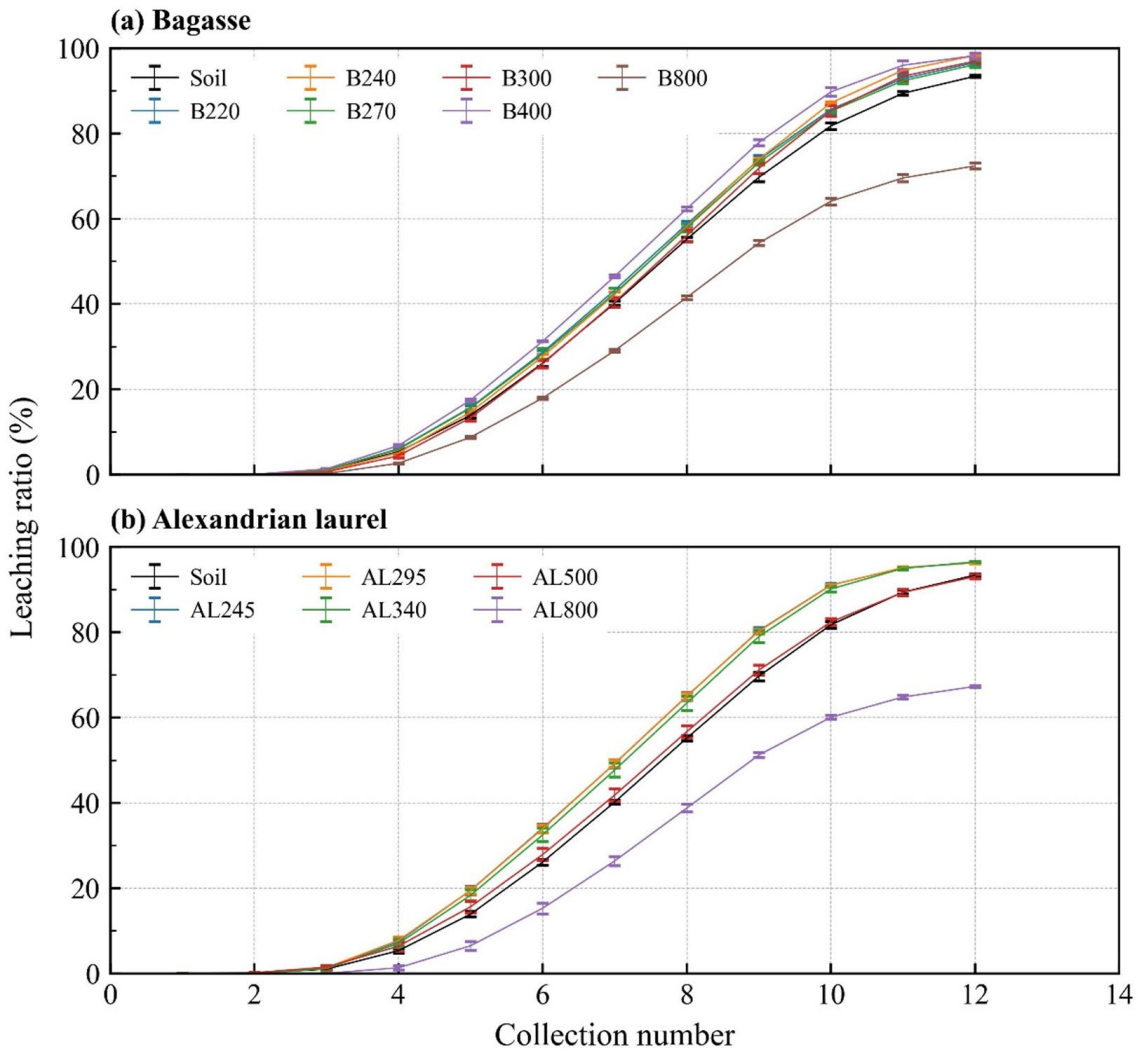
Changes in the nitrate leaching ratio. The ratio was obtained by dividing leaching nitrate by total applied nitrate (60 mg N). Error bars indicate standard error. B and AL indicate materials made from bagasse and Alexandrian laurel, respectively. The numbers after B or AL indicate the treatment temperature.

The pH of the mixed soil for the nitrate leaching test was almost the same as that of the soil (pH = 5.25); the pH range was 5.13–5.59 (Table 4). Each drainage volume was approximately 100 mL after each KNO_3_ solution or deionized water application; this was the same volume as applied KNO_3_ or deionized water. The saturated hydraulic conductivities were of the same order among the treatments (2.8 × 10^− 3^–9.0 × 10^− 3^ cm s^− 1^) and did not affect the drainage. Both bagasse- and Alexandrian-laurel-derived materials showed a reduction in leaching only in the 800 °C biochar with a mitigation rate of 30% (Fig. 5 and Supplementary Table S1; *p* < 0.01). Significant differences were observed at 312 min after the experiment with 800 °C bagasse and Alexandrian laurel biochar (*p* < 0.01). For the other temperatures, except for the 500 °C Alexandrian laurel material, leaching increased. For bagasse, a significant increase was detected at 345–378 min, and at the end, a significant increase was observed under all treatments (*p* < 0.01). For the Alexandrian laurel, a significant increase was observed at 279–312 min, and ultimately, the *p*-value was < 0.01. Only the 500 °C Alexandrian laurel material showed no significant difference from the soil.

**Table 4:** pH of soil-material mixture in leaching test.

B220	B240	B270	B300	B400	B800	AL245	AL295	AL340	AL500	AL800
5.25	5.38	5.23	5.25	5.25	5.38	5.14	5.14	5.13	5.45	5.59

B and AL indicate materials made from bagasse and Alexandrian laurel, respectively. The numbers after B or AL indicate the treatment temperature.

We attempted to determine the factors that affect the amount of nitrate leaching. The Gompertz model was applied to the leaching test results to determine the time (b/c) required to reach the maximum leaching rate. The parameters are presented in Supplementary Table S2; the R^2^ for all conditions was 0.999. The b/c at 800 °C was longer than that without application, whereas it was shorter at the other temperatures. In the multiple regression analysis, b/c was used as the objective variable, and EC and surface area were selected, yielding a significant regression equation with adjusted R^2^ = 0.96. The p-value for both parameters was < 0.01 and that for the surface area was smaller. This indicated that the surface area had a relatively strong effect on the b/c. The EC of all the materials was higher than that of the soil (Tables 1 and 2), although the b/c of the materials < 500 °C was shorter than that of the soil (Supplementary Table S2). The EC for 800 °C material was the highest among the others, whereas the b/c was the smallest. This relationship may be the reason why EC was selected in the multiple regression, However, biochar’s porous structure traps nitrate through pore-filling and steric entrapment^16^. Therefore, we considered the surface area to be one of the primary factors affecting the b/c. Under low-temperature conditions, the b/c of Alexandrian laurel was lower than that of bagasse, possibly owing to its hydrophilicity being lower than that of bagasse, which was indicated by the H/C ratio (Table 2). Although the pH of the mixed soil was not selected in the regression, we considered that it would play a role. In contrast with the pH of the adsorption test and materials (Tables 2 and 3a), the pH of mixed soil was low (< 6.0). A previous research study indicates that low pH increased nitrate adsorption^44^. Particularly, the materials that were treated at temperatures exceeding 400 °C were in a low pH environment owing to their application to soil; this differed from the adsorption test. This low pH would also mitigate nitrate leaching. The adsorption and leaching tests also indicated that low temperature material adsorbed nitrate but it was easy to move, that is, the retention was weak possibly owing to the small surface area.

### Practical implications and future directions

**Fig. 6:**
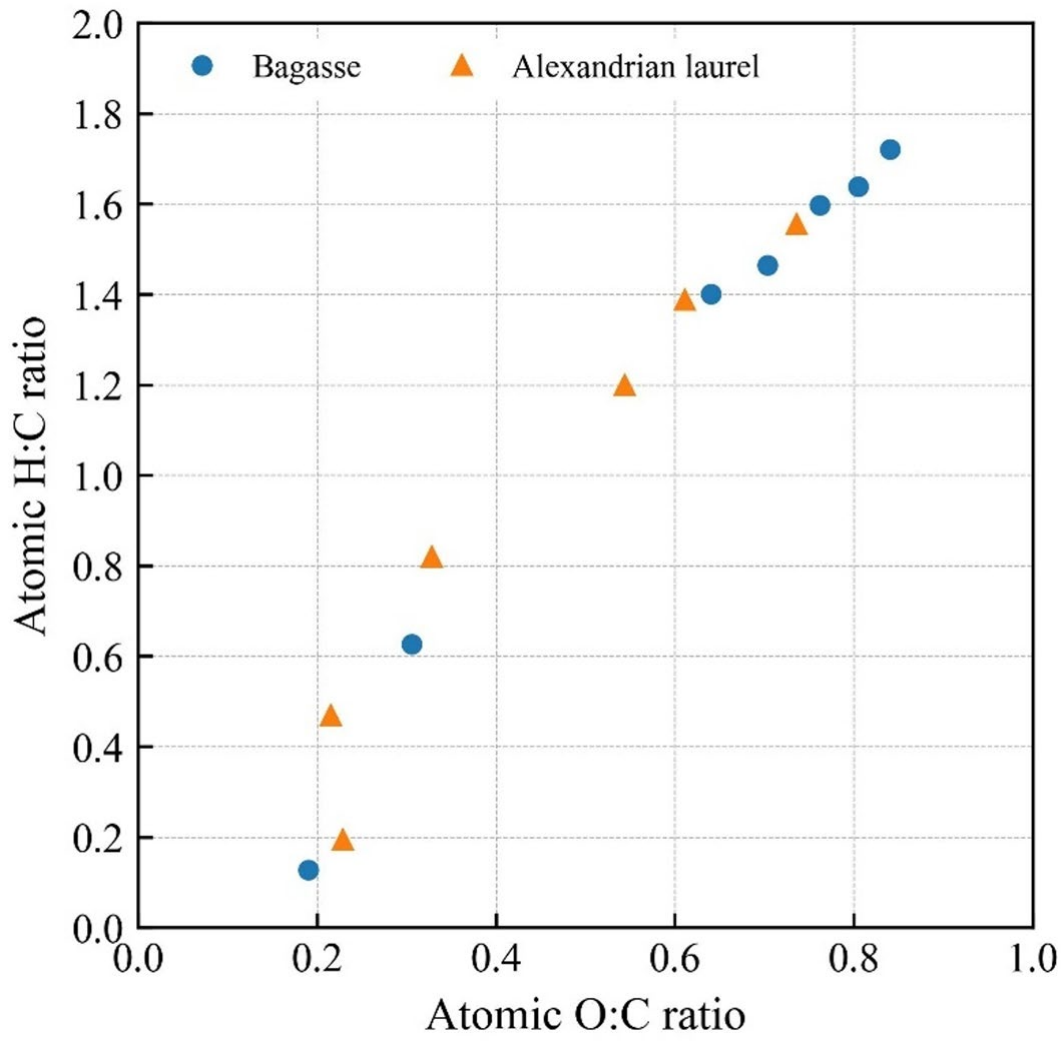
Atomic H: C and O: C ratios of all the materials. Atomic H: C ratio = (H content/1)/(C content/12). Atomic O: C ratio = (O content/16)/(C content/12).

In the present study, we evaluated a short-term effect of the thermally treated biomass. However, a long-term effect may differ. The atomic H: C and O: C ratios indicate the potential stability of materials, with lower ratios indicating a higher content of aromatic compounds and resistance of microbial degradation. The ratios decreased with increasing treatment temperature, indicating an increase in stability (Fig. 6 and Supplementary Table S3). The ratio drastically changed at > 300 °C. Both feedstock exhibited a lower ratio than that of the raw material after thermal treatment. Because the material at < 300 °C may have organic matter based on the FTIR (Fig. 2), the organic matter will remain in soil longer than the raw material. The possible remaining time would be six times longer than that of the raw material, based on the study by Itoh et al.^45^, who treated Japanese cedar sapwood with superheated steam at a temperature of up to 260 °C and evaluated their decay resistance using the outdoor exposed condition experiment. The slow decompose organic matter in materials at < 300 °C may contribute to improving soil fertility on the island. A recent review indicated that torrefied biomass application improves nutrient availability in the soil^26^. This nutrient availability and decoy existent organic matter will positively affect soil fertility in subtropical and tropical regions. Additionally, aged biochar has different properties compared to those of fresh biochar; it is oxidized and chemically active, but its physical benefits such as enhanced porosity decline^46–48^. This aging effect will occur in torrefied biomass, thereby enhancing chemical activities. However, the effect of torrefied biomass application has not been evaluated in field conditions, thus hindering the elucidation of the long-term effect and establishing the application technique. This assessment should be conducted in future studies.

## Conclusion

The bagasse- and Alexandrian-laurel-derived materials showed typical changes in the yield and functional groups, indicating that organic matter remained in materials treated below 300 °C. An increase in the nitrate adsorption rate was observed at 200–300 °C materials at a rate of approximately 3.0–7.0%. Nitrate leaching decreased only with the 800 °C material, with a mitigation rate of 30%. The experiments and multiple regression analysis revealed that a low pH and hydrophilicity of lower-temperature materials contributed to the high nitrate adsorption rate, whereas a large surface area of the material affected the nitrate leaching. Materials at < 500 °C did not mitigate nitrate leaching; some materials even accelerated the process, possibly because of low nitrate retention. The study evaluated a short-term effect of thermally treated biomass. However, the materials treated at < 300 °C retained organic matter, which was more stable than that in raw feedstock, ant will have different effects on nitrate leaching, soil fertility, and crop growth in the long term. Further investigation of the long-term effects of thermally treated biomass application at different treatment temperatures is necessary for in-depth knowledge on such topics and for spreading the technique in subtropical and tropical islands.

## Supplementary Information

Below is the link to the electronic supplementary material.

Supplementary Material 1

## Data Availability

All data supporting the findings of this study are available within the paper and its Supplementary Information.
