# Supplementary material for: Properties and nitrate leaching mitigation effect of thermally treated biomass-a case study of tropical and subtropical islands
Source: Sci Rep. 2026 Mar 3;16:11861. doi: 10.1038/s41598-026-41496-1 (PMC13066427; doi:10.1038/s41598-026-41496-1)
Supplement: Supplementary file 1 — Supplementary Material 1 [file 41598_2026_41496_MOESM1_ESM.docx]

**Supplementary information for**

**Properties and nitrate leaching mitigation effect of thermally treated biomass—a case study of tropical and subtropical islands**

Kosuke Hamada ^a,*^, Satoshi Nakamura ^b^, Takahiro Yoshida ^c^

^a^ Tropical Agriculture Research Front, Japan International Research Center for Agricultural Sciences, Okinawa, Japan

^b^ Crop, Livestock and Environmental Division, Japan International Research Center for Agricultural Sciences, Tsukuba, Japan

^c^ Forest Research and Management Organization, Tsukuba, Japan

^*^ Corresponding author, E-mail: [hamadak0454@jircas.go.jp](mailto:hamadak0454@jircas.go.jp)

**Supplementary Table S1.** Changes in the nitrate leaching ratio

| Collection number | Soil | B220 | B240 | B270 | B300 | B400 | B800 |
| --- | --- | --- | --- | --- | --- | --- | --- |
| 1 | 0.0± 0.0 | 0.0± 0.0 | 0.0± 0.0 | 0.0± 0.0 | 0.0± 0.0 | 0.0± 0.0 | 0.0± 0.0 |
| 2 | 0.1± 0.0 | 0.0± 0.0 | 0.0± 0.0 | 0.0± 0.0 | 0.0± 0.0 | 0.1± 0.0 | 0.0± 0.0 |
| 3 | 1.0± 0.2 | 1.1± 0.1 | 0.8± 0.2 | 1.0± 0.3 | 0.6± 0.1 | 1.3± 0.1 | 0.2± 0.0 |
| 4 | 5.4± 0.5 | 6.1± 0.3 | 5.1± 0.6 | 5.9± 0.9 | 4.4± 0.5 | 6.8± 0.3 | 2.6± 0.1 |
| 5 | 13.9± 0.6 | 15.7± 0.5 | 14.7± 0.7 | 15.6± 1.3 | 13.3± 0.8 | 17.5± 0.3 | 8.7± 0.2 |
| 6 | 26.1± 0.7 | 28.7± 0.5 | 27.7± 0.6 | 28.3± 1.3 | 26.0± 0.9 | 31.3± 0.1 | 17.9± 0.2 |
| 7 | 40.2± 0.5 | 43.3± 0.5 | 42.2± 0.6 | 42.6± 1.1 | 40.4± 1.1 | 46.5± 0.3 | 29.1± 0.3 |
| 8 | 55.2± 0.6 | 58.7± 0.7 | 58.2± 0.5 | 57.9± 0.9 | 56.1± 1.3 | 62.4± 0.4 | 41.5± 0.4 |
| 9 | 69.7± 1.0 | 73.8± 1.0 | 74.0± 0.3 | 73.2± 0.6 | 71.8± 1.3 | 77.9± 0.7 | 54.3± 0.6 ^**^ |
| 10 | 81.8± 0.8 | 85.7± 1.1 | 87.2± 0.3 ^*^ | 85.2± 0.2 | 85.3± 1.3 | 89.8± 1.0 ^**^ | 64.1± 0.8 ^**^ |
| 11 | 89.4± 0.4 | 92.9± 0.9 ^*^ | 94.8± 0.2 ^**^ | 92.4± 0.7 ^*^ | 93.4± 1.1 ^**^ | 96.0± 1.0 ^**^ | 69.6± 0.8 ^**^ |
| 12 | 93.4± 0.3 | 96.6± 0.9 ^**^ | 98.3± 0.2 ^**^ | 96.2± 0.8 ^**^ | 97.0± 0.5 ^**^ | 98.3± 0.7 ^**^ | 72.4± 0.7 ^**^ |

| Collection number | AL245 | AL295 | AL340 | AL500 | AL800 |
| --- | --- | --- | --- | --- | --- |
| 1 | 0.0± 0.0 | 0.0± 0.0 | 0.0± 0.0 | 0.0± 0.0 | 0.0± 0.0 |
| 2 | 0.0± 0.0 | 0.0± 0.0 | 0.0± 0.0 | 0.2± 0.1 | 0.0± 0.0 |
| 3 | 1.2± 0.4 | 1.4± 0.4 | 1.1± 0.3 | 1.5± 0.5 | 0.0± 0.0 |
| 4 | 7.5± 0.9 | 7.8± 0.8 | 7.0± 1.0 | 6.4± 1.1 | 1.3± 0.5 |
| 5 | 19.5± 1.0 | 19.4± 0.9 | 18.3± 1.4 | 15.6± 1.3 | 6.5± 1.1 |
| 6 | 34.1± 0.9 | 33.9± 0.9 | 32.5± 1.6 | 27.9± 1.4 | 15.3± 1.3 |
| 7 | 49.2± 1.0 | 49.2± 0.9 | 47.7± 1.6 | 41.8± 1.5 | 26.4± 1.1 |
| 8 | 65.0± 0.9 | 65.1± 0.8 ^*^ | 63.4± 1.7 | 56.7± 1.4 | 38.8± 0.9 |
| 9 | 80.4± 0.7 ^*^ | 80.3± 0.5 ^**^ | 79.0± 1.4 ^**^ | 71.1± 1.2 | 51.2± 0.6 ^**^ |
| 10 | 91.1± 0.5 ^**^ | 91.0± 0.2 ^**^ | 90.2± 0.6 ^**^ | 82.4± 0.9 | 60.1± 0.5 ^**^ |
| 11 | 95.1± 0.2 ^**^ | 95.2± 0.2 ^**^ | 95.0± 0.2 ^**^ | 89.4± 0.8 | 64.8± 0.4 ^**^ |
| 12 | 96.3± 0.1 ^**^ | 96.3± 0.2 ^**^ | 96.5± 0.1 ^**^ | 93.1± 0.5 | 67.3± 0.2 ^**^ |

The unit is %. The ratio was obtained by dividing leaching nitrate by total applied nitrate (60 mg N). ± indicates the standard error. Dunnett test was conducted for each time, and * and ** indicate significant differences (*p*<0.05 and *p*<0.01, respectively) compared to the soil. B and AL indicate materials made from bagasse and Alexandrian laurel, respectively. The numbers after B or AL indicate the treatment temperature.

**Supplementary Table S2.** Parameters of the Gompertz model

|  | **a** | **b** | **c** | **ac/exp** | **b/c** |
| --- | --- | --- | --- | --- | --- |
| Soil | 109.6±0.4 | 2.8±0.02 | 0.012±0.00 | 0.468±0.00 | 244.4±1.7 |
| B220 | 112.1±1.1 | 2.8±0.01 | 0.012±0.00 | 0.486±0.01 | 239.5±1.3 |
| B240 | 115.0±0.5 | 2.9±0.06 | 0.012±0.00 | 0.502±0.01 | 243.7±1.2 |
| B270 | 111.9±1.4 | 2.8±0.06 | 0.012±0.00 | 0.483±0.01 | 240.5±3.8 |
| B300 | 114.2±0.7 | 2.9±0.07 | 0.012±0.00 | 0.498±0.01 | 247.3±2.8 |
| B400 | 112.4±0.6 | 2.9±0.05 | 0.012±0.00 | 0.510±0.01 | 233.0±0.2 |
| B800 | 83.7±0.9 | 3.1±0.03 | 0.013±0.00 | 0.390±0.01 | 247.9±1.2 |
| AL245 | 107.8±0.7 | 3.0±0.07 | 0.013±0.00 | 0.523±0.01 | 223.6±2.5 |
| AL295 | 107.9±0.5 | 2.9±0.06 | 0.013±0.00 | 0.521±0.01 | 223.7±2.1 |
| AL340 | 108.8±0.5 | 2.9±0.04 | 0.013±0.00 | 0.518±0.00 | 227.6±3.9 |
| AL500 | 108.5±0.4 | 2.8±0.08 | 0.012±0.00 | 0.463±0.01 | 239.3±3.7 |
| AL800 | 75.6±0.7 | 3.5±0.18 | 0.014±0.00 | 0.394±0.02 | 247.4±3.3 |

a: maximum potential leaching rate, b: intercept, c: leaching rate. The actual maximum leaching rate during the experiment was determined by ac/exp and the time (min) at this point was determined by b/c. B and AL indicate materials made from bagasse and Alexandrian laurel, respectively. The numbers after B or AL indicate the treatment temperature.

**Supplementary Table S3.** Atomic H:C and O:C ratios of each material

|  | Atomic H:C ratio | Atomic O^:^C ratio |
| --- | --- | --- |
| Bagasse | 1.72 | 0.84 |
| B220 | 1.64 | 0.80 |
| B240 | 1.60 | 0.76 |
| B270 | 1.47 | 0.70 |
| B300 | 1.40 | 0.64 |
| B400 | 0.63 | 0.31 |
| B800 | 0.13 | 0.19 |
| Alexandrian laurel | 1.56 | 0.74 |
| AL245 | 1.39 | 0.61 |
| AL295 | 1.20 | 0.54 |
| AL340 | 0.82 | 0.33 |
| AL500 | 0.47 | 0.21 |
| AL800 | 0.19 | 0.23 |

B and AL indicate materials made from bagasse and Alexandrian laurel, respectively. The numbers after B or AL indicate the treatment temperature. Atomic H:C and O:C ratios were calculated by (H content/1) / (C content/12) and (O content/16) / (C content/12), respectively.
